# Puerperal Fever in Cook Co. Hospital

**Published:** 1870-05

**Authors:** 


### Chicago Medical Journal.

A MONTHLY RECORD OF

*Medicine, Surgery, and the Collateral Sciences.*

EDITED BY J. ADAMS ALLEN, M.D., LL.D., AND WALTER HAY, M.D.

---

VOL. XXVII. — MAY, 1870. — NO. 5.

---

### Original Communications.

ARTICLE I. — *Puerperal Fever in Cook County Hospital.*

The following is a report of a Committee appointed at the regular meeting of the Medical Board of the Hospital, January 31st, 1870, to investigate the causes and management of the recent epidemic of Puerperal Fever in this Hospital, which was read at the regular meeting of the Board, February 28th, 1870, and ordered published in the medical journals of this city, and furnished to the Chicago Medical Society.

T. D. FITCH, M. D., Sec'y Med. Board Cook Co. Hospital.

*To the Medical Board of Cook County Hospital:*

GENTLEMEN: The undersigned, appointed a committee at your last meeting to investigate the management of the lying-in wards of the Hospital during the prevalence of the late epidemic of Puerperal Fever, beg leave to report: That the resources of our art for the prevention, and in the presence of such epidemic manifestations, may be briefly stated as the following:

1st. Cleanliness of person, and clothing, and apartments of the lying-in women; ventilation of the wards; disinfection and removal of any sources of putrid effluvia; avoidance of the results of over-crowding; a properly regulated temperature; and appropriate alimentation.

2nd. These means failing to prevent the occurrence of cases of the disease, in addition to them, it now becomes necessary to isolate the patients as promptly and completely as possible, to make the disinfection and ventilation, if possible, yet more thorough; and on failure of these means to arrest the disease among the parturient patients,

3d. It then becomes necessary to vacate the wards, and refuse admission to this class of patients, experience proving that the risk is too great to continue the occupancy.

4th. The disuse of the wards should be for such length of time and seconded by such renovation, cleansing and disinfection as experience proves most efficient in thoroughly removing any real or suspected sources of infection from the wards themselves, the walls, ceiling, floors, etc., and all they may contain of clothing, furniture, etc. The treatment of such cases as do occur, notwithstanding these precautions, is so well settled that it is unnecessary to refer to it in detail; suffice it to say, that the indications to be filled by sedatives, counter-irritants and opiates, and in toxæmic cases eliminants, with such special modifications as particular complications demand, finding their indications in sound pathology and therapeutics, is the practice of the most authoritative names in gynæcological science. In the light of these principles your committee has thoroughly investigated the facts in regard to the late epidemic prevalent in the Hospital, and find they have been fully and faithfully observed. That scrupulous cleanliness, thorough disinfection, isolation of the patients, so far as practicable, and vacation of the wards, were all effected at such times as were proper and necessary. That the whole house has been thoroughly white-washed twice, and the lying-in wards three times, since these cases began to appear. That such disinfectants as chloride of lime, carbolic acid, spray of permanganate of potassa, and solution of chlorinated soda, have been freely and persistently used. That the methods of treatment put in practice, were such as have already been noted, and that in their results, the most favorable effects were witnessed, notwithstanding formidable obstacles, which we beg leave to bring to your notice. It is well known by all familiar with the classes of lying-in patients to be found in a pauper hospital, that they are, from the first, in notoriously unfavorable conditions for a safe and normal termination of gestation. The immense majority being either the unfortunate victims of seduction, abandoned to hardship and exposure, depressed and debilitated by moral and physical causes, more or less intense and long continued, or women of abandoned and depraved habits of life, intemperate, and exhausted by venereal excesses and diseases, arriving at the hospital as a last resort, having failed of all other means of existence; while it is the exception and not the rule to meet with women in average favorable moral and physical conditions for the act of childbirth. Under these circumstances, if we add to the gravity of this dangerous disease these unfavorable conditions, we should naturally expect a very high mortality, even with the most skillful treatment, which your committee is happy to announce, in this instance, as being much less than could reasonably have been anticipated.

In effect, from August, 1869, to February, 1870, the period during which the epidemic influence has principally made itself felt, the number of cases of puerperal fever occurring was sixteen, of which ten recovered and six died; a lower death rate than can be shown, under like conditions, in any published report with which your committee is familiar.

And your committee would further and finally report, after careful investigations, that any representations of neglect or mismanagement of this department of the hospital, can owe their origin to only one of two circumstances, viz: ignorance of facts or their willful falsification.

All of which is respectfully submitted.

THOS. BEVAN, *Chairman.*

W. H. BYFORD.

EDWIN POWELL.

| | |
|---|---|
| W. H. Byford, | R. C. Hamill, |
| J. W. Freer, | J. R. Gore, |
| H. A. Johnson, | Jos. P. Ross, |
| Thomas Bevan, | Edwin Powell, |
| R. G. Bogue, | Chas. G. Smith, |
| H. M. Lyman, | H. W. Jones, |
| T. D. Fitch, | Jas. S. Hildreth, |

*Board of Cook Co. Hospital.*

